# A Survey on Orbital Space-Occupying Lesions during a Twelve-Year Period from a Referral Center in Iran

**DOI:** 10.18502/jovr.v18i2.13187

**Published:** 2023-04-19

**Authors:** Abbas Bagheri, Parisa Ashtar-nakhaie, Maryam Aletaha, Bahareh Kheiri, Amirreza Veisi

**Affiliations:** ^1^Ocular Tissue Engineering Research Center, Research Institute for Ophthalmology and Vision Science, Shahid Beheshti University of Medical Sciences, Tehran, Iran; ^2^Ophthalmic Research Center, Research Institute for Ophthalmology and Vision Science, Shahid Beheshti University of Medical Sciences, Tehran, Iran

**Keywords:** Benign Tumor, Extraconal Tumor, Intraconal Tumor, Malignant Tumor, Orbital Tumor

## Abstract

**Purpose:**

In this study, we describe different orbital space-occupying lesions (SOLs) from a referral center in Iran.

**Methods:**

In this retrospective case series, all records of “orbital tumors” with a definite histopathologic diagnosis at a referral center in Iran were reviewed from April 2008 to May 2020.

**Results:**

A total of 375 orbital SOLs were included. The study population consisted of 212 (56.5%) female and 163 (43.5%) male subjects with overall mean age of 31.09 ± 21.80 years. The most common clinical presentation was proptosis and the superotemporal quadrant was the most frequent site of involvement. Extraconal lesions (276 cases, 73.6%) outnumbered intraconal lesions (99 cases 26.4%). The great majority of SOLs (344, 91.7%) were primary, while 24 (6.4%) were secondary and 7 (1.9%) were metastatic. Benign lesions (309, 82.4%) were much more common than malignant SOLs (66, 17.6%). Overall, dermoid cysts and malignant lymphoma were the most prevalent benign and malignant orbital SOLs, respectively. The malignant to benign lesion ratio was 0.46 in children ( ≤ 18 years), 0.81 in middle-aged subjects (19–59 years), and 5.9 in older ( ≥ 60 years) cases. The most common type of malignancy was rhabdomyosarcoma in children, lymphoma in middle-aged subjects, and invasive basal cell carcinoma in older age group.

**Conclusion:**

Over the 12-year study period, benign, primary, extraconal orbital SOLs were more frequent than malignant, secondary, and intraconal lesions. The ratio of malignant lesions increased with age in this cohort of patients.

##  INTRODUCTION

The orbit is a compact area which is comprised of different tissues including bone, fat, vascular, neural, and muscular components, each of which may be afflicted with various benign and malignant space-occupying lesions (SOLs). SOLs may originate from vestigial tissues or may be the result of invasion from periorbital spaces such as paranasal sinuses, nasal cavity, intracranial space, or even metastasis from distant areas.^[1]^ Orbital SOLs have different presentations ranging from trivial cosmetic issues to loss of sight or even life-threatening conditions.^[2]^

The frequency of various orbital SOLs varies according to geographic area, ethnicity, age, and sex. This frequency also depends on the department where the study originates from; results from an oculoplastic department may differ from those of a neurosurgery or otolaryngology department.^[2,3,4,5,6]^ Diagnostic criteria are also critical and influence the results of studies; reports based on pathologically confirmed specimens are entirely different from studies that are limited to clinical or radiological diagnoses.^[2,3,4,5,6][7]^

Herein we provide data based on histopathologically confirmed orbital SOLs over a 12-year period from an oculoplastic referral hospital in Iran.

##  METHODS 

This retrospective study was based on data from records of patients registered with a general diagnostic code of “orbital tumor” from April 2008 to May 2020 at the Oculoplastic Service of the Ophthalmology Department at Labbafinejad Medical Center. The study was approved by the ethics committees of the Ophthalmic Research Center affiliated with Shahid Beheshti University of Medical Sciences; the approval number was IR.SBMU.ORC.REC.1399.008. The study adhered to the Declaration of Helsinki and informed written consent was obtained from all patients or their guardians prior to surgery.

In all included subjects, orbital SOLs had undergone either incisional or excisional biopsy, and a confirmed histopathologic diagnosis had been established. All cases with unconfirmed pathology were excluded from the study even if a clinical or radiological diagnosis was available.

Demographic data, including age, sex, and laterality of involvement, were determined. Topographic involvement of the orbit was also documented according to results of imaging studies including CT scan and/or MRI. As much as the results of the general physical examinations were documented in the files, these data were also recorded.

Based on definite histopathologic diagnosis, orbital SOLs were classified into seven principal groups including cystic lesions, vascular lesions, neurogenic lesions, inflammatory and lymphoproliferative lesions, mesenchymal lesions, epithelial lacrimal gland lesions, and secondary or metastatic lesions. Notably, inflammatory lesions were included only if they had produced a mass lesion. Lymphoproliferative and inflammatory lesions of the lacrimal gland were tabulated twice: first in the subgroup of inflammatory and lymphoproliferative lesions and a second time under lacrimal gland lesions; however, they were counted only once. We also classified each major group into subgroups, differentiated benign lesions from malignant lesions, and determined the relative frequencies of such lesions according to age.

We used frequency (percentage), mean values, median, and range to describe the data. Statistical analyses were performed using the SPSS software (Version 25.0 Released 2017, IBM SPSS Statistics for Windows, IBM Corp. Armonk, NY, USA).

**Table 1:** Demographic characteristics of patients and space-occupying lesions.


**Specificity**	**Categories**	**Patients number (%)**
Sex	Female	212 (56.5)
	Male	163 (43.5)
Laterality	Left	190 (50.7)
	Right	185 (49.3)
Position	Extraconal	276 (73.6)
	Intraconal	99 (26.4)
Origination	Primary	344 (91.7)
	Secondary	24(6.4)
	Metastatic	7(1.9)
Pathology	Benign	309 (82.4)
	Malignant	66 (17.6)
Age range (yr)	Children ( ≤ 18)	123 (32.8)
	Middle aged (19–59)	209 (55.7)
	Old aged ( ≥ 60)	43 (11.5)
	
	

**Table 2:** Major subclassifications of orbital space-occupying lesions in order of their frequencies, together with their age and sex distribution.


**Major pathologies**	**Number (%)**	**Mean age ± SD**	**Female/Male**
Cystic	138 (36.8)	15.89 ± 14.24	82/56
Vascular	88 (23.5)	35.18 ± 16.79	53/35
Inflammatory and lymphoproliferative	40 (10.7)	45.00 ± 19.38	22/18
Secondary and metastatic	31 (8.2)	60.19 ± 18.79	16/15
Neurogenic	29 (7.7)	36.65 ± 14.77	20/9
Mesenchymal	28 (7.5)	26.29 ± 22.71	13/15
Epithelial lesions of lacrimal gland	21 (5.6)	43.14 ± 20.25	6/15
Total	375 (100)	31.09 ± 21.80	212/163
	
	
SD, standard deviation			

**Table 3:** Cystic orbital space-occupying lesions.


**Cystic lesions**	**Number of Patients (%)**	**Percentage of total**
Dermoid cyst	113 (82.0)	30.1
Ductul lacrimal gland cyst	7 (5.1)	1.8
Epidermoid cyst	4 (2.9)	1.1
Epithelial cyst (inclusion cyst)	4 (2.9)	1.1
Mucocele	4 (2.9)	1.1
Cystic teratoma	2 (1.4)	< 1
Hydatid cyst	2 (1.4)	< 1
Hematic cyst	1 (0.7)	< 1
Fibroadipose vascular anomaly cyst	1 (0.7)	< 1
Total	138 (100)	36.8
	
	

**Table 4:** Vascular orbital space-occupying lesions.


**Vascular lesions**	**Number of patients (%)**	**Percentage of total**
Cavernous hemangioma	66 (75.0)	17.6
Lymphangioma	13(14.9)	3.4
Hemangiopericytoma	4 (4.5)	1.1
Capillary hemangioma	2 (2.3)	< 1
Angiosarcoma	1 (1.1)	< 1
Angioleiomyoma	1 (1.1)	< 1
Arteriovenous malformation	1 (1.1)	< 1
Total	88 (100)	23.5
	
	

**Table 5:** Inflammatory and lymphoproliferative orbital space-occupying lesions.


**Inflammatory & lymphoproliferative lesions**	**Number of patients (%)**	**Percentage of total**
Pseudotumor	17 (42.5)	4.5
Lymphoma	16 (40.0)	4.3
Lymphoid hyperplasia of lacrimal gland	7 (17.5)	1.9
Total	40 (100)	10.7
	
	

**Table 6:** Mesenchymal orbital space-occupying lesions.


**Mesenchymal lesions**	**Number of patients (%)**	**Percentage of total**
Rhabdomyosarcoma	8 (28.6)	2.1
Dermolipoma	7 (25.0)	1.9
Xanthogranuloma	5 (17.9)	1.3
Fibroma	2 (7.1)	< 1
Liposarcoma	2 (7.1)	< 1
Osteoma	2 (7.1)	< 1
Giant cell tumor of bone	1 (3.6)	< 1
Chondrosarcoma	1 (3.6)	< 1
Total	28 (100)	7.5
	
	

**Table 7:** Neurogenic orbital space-occupying lesions.


**Neurogenic lesions**	**Number of patients (%)**	**Percentage of total**
Meningioma	11 (37.9)	2.9
Schwannoma	9 (31.0)	2.4
Neurofibroma	8 (27.7)	2.1
Glioma	1 (3.4)	< 1
Total	29 (100)	7.7
	
	

**Table 8:** Secondary and metastatic orbital space-occupying lesions.


**Secondary and metastatic**	**Number of patients (%)**	**Percentage of total**
BCC from eyelids	11 (35.5)	2.9
SCC from eyelids	5 (16.1)	1.3
Breast carcinoma	3 (9.7)	< 1
Choroidal melanoma	2 (6.5)	< 1
Sebaceous cell carcinoma	2 (6.5)	< 1
Ewing sarcoma	2 (6.5)	< 1
SCC from conjunctiva	1 (3.2)	< 1
Leukemia	1 (3.2)	< 1
Fibrosarcoma from paranasal sinuses	1 (3.2)	< 1
Multiple myeloma	1 (3.2)	< 1
Melanoma from skin	1 (3.2)	< 1
Melanoma from conjunctiva	1 (3.2)	< 1
Total	31 (100)	8.2
	
	
BCC, basal cell carcinoma; SCC, squamous cell carcinoma		

**Table 9:** Lacrimal gland space-occupying lesions.


**Lacrimal gland lesions**	**Number of patients (%)**			**Percentage of total**
Epithelial lesions 21 (55.3) 5.6				
Pleomorphic adenoma		11 (28.9)	2.9	
Adenoid cystic carcinoma		8 (21.1)	2.1	
Malignant mixed tumor (pleomorphic adenocarcinoma)		2 (5.3)	< 1	
Lymphoproliferative lesions		10 (26.3)	2.6	
Benign lymphoid hyperplasia		7 (18.4)	1.9	
Lymphoma		3 (7.9)	< 1	
Ductal lacrimal gland cyst		7 (18.4)	1.9	
Total		38 (100)	10.1	
	
	

**Table 10:** The most prevalent orbital space-occupying lesions.


**Orbital lesion**	**Number**	**Percentage of total**
Dermoid cyst	113	30.1
Cavernous hemangioma	66	17.6
Pseudotumor	17	4.5
Lymphoma	16	4.3
Lymphangioma	13	3.4
Pleomorphic adenoma	11	2.9
BCC from eyelid	11	2.9
Schwannoma	9	2.4
Rhabdomyosarcoma	8	2.1
Total	264	70.2
	
	

**Table 11:** Stratification of the most prevalent benign and malignant orbital space-occupying lesions according to age.


**Orbital lesions**	** ≤ 18 years**	**19–59 years**	** ≥ 60 years**
Benign (numbers) Dermoid cyst (113)	73	39	1
Cavernous hemangioma (66)	4	56	6
Pseudotumor (17)	3	13	1
Pleomorphic adenoma (11)	1	6	4
Lymphangioma (13)	10	3	0
Schwannoma (9)	2	7	0
Others (80)	19	54	7
309 (82.4% of total)	112 (36.2% of benign)	178 (57.6% of benign)	19 (6.2% of benign)
Malignant (numbers)		
Lymphoma (16)	0	10	6
BCC from eyelids (11)	0	1	10
Rhabdomyosarcoma (8)	8	0	0
Adenoid cystic carcinoma (8)	0	7	1
SCC from eyelids (5)	0	2	3
Breast carcinoma (3)	0	2	1
Others (15)	3	9	3
66 (17.6% of total)	11 (16.7% of malignant)	31 (46.9% of malignant)	24 (36.4% of malignant)
	
	

**Table 12:** Location of orbital space-occupying lesions.


**Orbital location**	**Number of patients**	**Percentage of total**
Superotemporal	123	32.8
Intraconal	99	26.4
Nasal	50	13.3
Diffuse	48	12.8
Superonasal	14	3.7
Inferior	13	3.5
Superior	13	3.5
Temporal	6	1.6
Inferonasal	6	1.6
Inferotemporal	3	0.8
Total	375	100
	
	

**Table 13:** Signs and symptoms of patients.


** Symptom or sign**	**Number of patients**	**Percentage of total**
Proptosis	159	42.4
Lump	119	31.7
Ocular displacement	61	16.2
Ptosis	34	9.1
Lid shape deformity	25	6.7
Conjunctival injection	13	3.5
Pain	12	3.2
Lid retraction	6	1.6
Conjunctival chemosis	6	1.6
Conjunctival mass	5	1.3
Diplopia	5	1.3
	
	

##  RESULTS

The study population consisted of 375 patients, including 212 (56.5%) female and 163 (43.5%) male subjects with a mean age of 31.09 ± 21.80 (range: 1–94) years. The mean age was 29.0 ± 20.85 (range: 1–87) years in female patients and 33.9 ± 22.7 (range: 1–94) years in male subjects. We encountered no bilateral involvement in this study, and the right and left sides were almost equally involved. Overall, out of the 375 SOLs, 309 lesions (82.4%) were benign and 66 (17.6%) were malignant; 276 lesions (73.6%) were extraconal while 99 (26.4%) were intraconal; 344 (91.7%) were primary lesions, 24 (6.4%) were secondary, and 7 (1.9%) were metastatic lesions [Table 1].

The number and percentage of patients in major groups with mean age at presentation and gender ratios are summarized in Table 2; corresponding data for subgroups are presented in Tables 3–9. The most prevalent orbital SOLs, irrespective of benign or malignant nature, are presented in Table 10, and the most prevalent benign and malignant lesions stratified by age are shown in Table 11. The most common category of lesions in this study was cystic lesions and the most common benign lesion was dermoid cyst. The leading malignant lesion was lymphoma, the most common secondary lesion was invasive eyelid basal cell carcinoma (BCC), and the most frequent metastatic lesion originated from breast carcinomas.

We categorized predominant benign and malignant lesions of the orbit according to age at presentation in Table 11. Benign lesions were more prevalent in lower age groups and the frequency of malignant lesions increased with age. The ratio of malignant to benign lesions was 0.46 in children ( ≤ 18 years), 0.81 in middle-aged patients (19–59 years), and 5.9 in older individuals ( ≥ 60 years).

The site of involvement is presented in Table 12 and demonstrates that the most common site of involvement was the superotemporal quadrant of the orbit. The leading presenting symptoms and signs are presented in Table 13. Proptosis was the most prevalent symptom in 42.4% of patients, while pain was only present in 3.2% of subjects in the current series.

##  DISCUSSION

This study evaluated all histopathologically confirmed orbital SOLs over a 12-year period from an oculoplastic referral center in Iran. Generally, there are two types of studies on the epidemiology of orbital SOLs in the medical literature. The first group categorizes the lesions according to the clinical and radiological findings; although such studies may be partly representative, the real findings are not definite. The second group of studies is based on a definite histopathologic diagnosis that suffers from omission of specific lesions which do not regularly undergo biopsy for a diagnosis, such as capillary hemangioma, optic nerve glioma, and meningioma. These second type of reports may also be representative of subjects who require surgical intervention because of functional or cosmetic issues.^[6,8]^

Our study showed that the primary lesions were 11.1 times more common than secondary and metastatic lesions. These observations are in line with other large series.^[2,3][6,8][9][10]^ In our series, benign orbital lesions outnumbered malignant lesions by 4.7 times which is comparable to the report by Kodsi et al,^[11]^ however, in some of the other studies this predominance of benign lesions was less significant,^[2][6,8][12]^ which may be due to the younger age of our study population.

Dermoid cysts were the most common lesions comprising one-third of all lesions in our study, which is similar to other large series;^[2,3]^ however, in the study by Ohtsuka et al, dermoid cysts were the fifth most common tumor.^[9]^ Cavernous hemangioma was the second most common tumor in our series, making up 17.6% of all lesions, which is comparable to some other comprehensive series.^[2,3][6][9][13]^

Lymphoma was the most common malignant tumor in our series, comprising 4.3% of all tumors, which is similar to findings by various alternate studies.^[7,14,15,16]^ It is notable that the incidence of lymphoma in some developed countries outweighs our rates by two to six times.^[4,5,9,17,18]^ As an example, in two reports by Shields et al;^[3,6]^ it was demonstrated that the rate of orbital lymphoma increased from 4% to 8% in the United States after 20 years which may reflect improvements in life expectancy, patient care, and diagnostic capabilities. Moslehi et al^[19]^ and Sjo et al^[20]^ have documented an increase in the incidence of non-Hodgkin lymphoma in orbit and adnexa with increasing age. Our study population was skewed toward younger subjects, and patients older than 60 years comprised only 11.5% of cases which may explain this relatively low rate.

The most common secondary orbital SOL in our report was invasive eyelid BCC which is compatible with the findings by Bonavolonta et al.^[2]^ Kennedy reported orbital invasion by eyelid skin tumors as the second common cause of secondary orbital tumors following invasive tumors from paranasal sinuses.^[8]^ Shields et al, in a report in 1984,^[3]^ noted orbital invasion by eyelid BCC and invasive choroidal melanoma as the most common secondary orbital tumors with an incidence of 3%. In another report by the same group of authors in 2004,^[6]^ invasive BCC ranked fourth with an incidence of only 1% being outnumbered by invasive uveal melanoma and invasive paranasal sinus tumors. Improved care and patient surveillance have probably decreased orbital invasion by eyelid skin malignancies, while more efficacious management for malignant uveal melanoma preserves the eye obviating the need for enucleation and possibly increasing the rate of orbital uveal melanoma. The incidence of orbital invasion by uveal melanoma in our series was only 0.5%. Some older reports from developing areas have revealed that orbital invasion by retinoblastoma accounts for more than half of all orbital SOLs in children.^[21,22]^ In the current series, although 32.8% of all subjects were < 18 years of age, we observed no case of orbital invasion by retinoblastoma which may reflect timely diagnosis and treatment of retinoblastoma cases in our population.

Metastatic lesions comprised 1.9% of all orbital SOLs in our study, which is compatible with other studies.^[6,8]^ In the two studies by Shields et al,^[3,6]^ the frequency of orbital metastasis increased from 2.5% to 4% over a 20-year period. Similar to our research, some large series on orbital SOLs have revealed breast carcinoma as the most common source of orbital metastasis.^[2,3][4][6][23][24]^ The two other primary malignancies which may be among the common causes for orbital metastasis are lung and prostate cancers; however, they were not detected in our series.^[2,3,4,5,6]^ It is notable that in various studies from Japan, orbital metastasis from lung carcinoma outnumbered alternative sites of origin.^[9,25]^

We observed that the prevalent benign lesion in subjects under 18 years of age was dermoid cyst which accounted for 73 out of 123 cases (59.3%) in this age group, followed by lymphangioma, which involved 10 out of 123 patients (8.1%). This is compatible with multiple prior studies on orbital tumors in children; however, capillary hemangioma was shown to outnumber lymphangioma in these studies as the evaluation was based on clinical data instead of histopathology.^[26,27,28]^ Orbital malignancies are usually rare in the pediatric age group overall;^[26,27]^ the most common malignant lesion in this age group was rhabdomyosarcoma which accounted for 8 out of 123 cases (6.5%) and was comparable to previous studies.^[2,26,28,29]^

The ratio of extraconal to intraconal lesions in our study was 2.8, which is consistent with other comparable studies reporting ratios ranging from 2 to 7.^[4,9,17]^ The most common topographic area of involvement in our series was the superotemporal quadrant of the orbit comprising about one-third of all cases. This finding is similar to an extensive study by Bonavolonta et al^[2]^ on 2480 patients with orbital tumors. In another study on a population older than 60 years, Demirci et al^[5]^ reported that the area of orbital involvement in 53% of cases was the superior orbit which is consistent with our study despite our younger population of subjects with a mean age of 30 years.

Our series included 38 cases of lacrimal gland lesions comprising 10.1% of all orbital tumors, which is line with previous reports.^[2,31]^ Epithelial lesions were about twice more common than lymphoproliferative lesions, although these entities are often reported equally.^[1]^ Pleomorphic adenoma and adenoid cystic carcinoma were the most common benign and malignant epithelial tumors of the lacrimal gland which is consistent with previous studies on lacrimal gland tumors.^[2,8,32]^ Benign reactive lymphoid hyperplasia and lacrimal gland ductal cyst were the two most common non-epithelial lesions of the lacrimal gland in our study, which differs from many large studies reporting chronic dacryoadenitis as the leading pathology in this category.^[3][6,8][33]^ We observed two cases of malignant mixed tumors, both of which were primary tumors and not the recurrence of pleomorphic adenoma. In most studies, this tumor is reported to be less prevalent than adenoid cystic carcinoma.^[2,3][4][6]^ In a review article,^[31]^ the rate of adenoid cystic carcinoma was 3.5 times that of pleomorphic adenocarcinoma.

The most common clinical presentations in our series were proptosis and lump sensation in 42.4% and 31.7% of patients, respectively. This is consistent with a study that recorded clinical findings,^[34]^ although in another study focusing on metastatic lesions, ocular motility problems was a more common symptom than mass effect.^[24]^ Some studies have reported bilaterality in 2–8% of their cases,^[5,17,25]^ however we did not encounter any bilateral cases which may be due to the paucity of metastatic lesions in our series.

This study suffers from referral bias and other drawbacks inherent to all retrospective studies. Since only biopsy-proven cases were selected, a small number of familiar entities such as capillary hemangioma, optic nerve glioma, and meningioma were recorded.

In summary, at our referral center, 91.7% of orbital SOLs were primary lesions, the most common orbital SOLs were cystic lesions, benign lesions were 4.7 times more common than malignant ones, and extraconal lesions were 2.8 times more common than intraconal lesions.

##  Financial Support and Sponsorship

None.

##  Conflicts of Interest

The authors have no financial interest in the subject of this article.
